# Sexual and gender based violence in Nigeria tertiary institutions: exploring the roles of stakeholders and best practices

**DOI:** 10.3389/fgwh.2026.1700008

**Published:** 2026-05-13

**Authors:** Oyeyemi Bukola Babalola, Lanre Ikuteyijo, Oluyemisi O. Obilade, Akanni Ibukun Akinyemi, Oluwatoyin Olatundun Ilesanmi, Bisola I. Adebayo

**Affiliations:** 1 Department of Psychology, Obafemi Awolowo University, Ife, Nigeria; 2 Department of Sociology and Anthropology, Obafemi Awolowo University, Ife, Nigeria; 3 Department of Adult Educationand Life-Long Learning, Obafemi Awolowo University, Ife, Nigeria; 4 Department of Demography and Social Statistics, Obafemi Awolowo University, Ife, Nigeria; 5 Center for Gender, Redeemer University, Ede, Nigeria; 6 Lagos State University Teaching Hospital, Lagos, Nigeria

**Keywords:** gender, sexual, stakeholders, tertiary institutions, violence

## Abstract

Sexual and Gender-Based Violence (SGBV) remains a major global public health and human rights concern with profound implications for women's safety, dignity, and quality of life. In Nigeria, SGBV is pervasive, particularly within tertiary institutions, where young women and adolescent girls face heightened vulnerability. This study explored the roles of key stakeholders and identified best practices in addressing SGBV within Nigerian higher education institutions to inform effective policies and interventions. A cross-sectional, sequential mixed-methods design was employed. Quantitative data were collected from 4,142 participants across tertiary institutions in Kaduna, Enugu, Osun, and the Federal Capital Territory (FCT) using multistage stratified random sampling. Qualitative data were obtained through 22 key informant interviews, 12 in-depth interviews, and 7 focus group discussions with relevant stakeholders. The study was anchored on the Socio-Ecological Model (SEM) and the Theory of Change (ToC), which facilitated a multi-level analysis of influences and linked diagnostic insights to actionable intervention pathways. Quantitative data were analyzed with SPSS version 25, while qualitative data were thematically analyzed using ATLAS.ti software. Findings indicated that most stakeholders addressing SGBV were non-state actors, including student associations (17.4%), parents (12.6%), academic staff (11.6%), and non-governmental organizations (13.5%). These groups were particularly active in Osun State (37.7%) and the FCT (18.3%). Qualitative findings corroborated these patterns, emphasizing the visible roles of NGOs and student-led organizations in awareness creation, advocacy, and survivor support. TFG, state actors such as the Ministry of Education (8.7%), law enforcement agencies (7.7%), the judiciary (4.8%), and institutional management (7.7%) were primarily recognized for policy formulation and enforcement, collectively accounting for 28.9% of total mentions. Although their formal authority was acknowledged, their operational presence within institutions appeared limited. Notably, effective collaborations observed in Osun and Abuja provided functional models of multi-stakeholder engagement and survivor-centered responses. The study concludes that SGBV prevention in Nigerian tertiary institutions is largely driven by non-state actors amid weak state engagement. Strengthening coordination, enforcing policies, and institutionalizing gender-responsive frameworks are critical to fostering sustainable, equitable, and violence-free campuses.

## 1 Background

Sexual and Gender-Based Violence (SGBV) is a global public health concern and a grave violation of human rights due to its far-reaching effects on victims' physical health, quality of life, and overall well-being, particularly among adolescent girls and young women. Gender-based violence (GBV) relates to any gender-based act that results in or is likely to result in physical, sexual, or mental harm or suffering to women, including threats of such acts, coercion, or arbitrary deprivation of liberty, whether occurring in public or in private life (Muluneh et al., 2019; 1). Sexual and Gender-Based Violence (SGBV) within higher education institutions represents both a public health crisis and a structural injustice that threatens academic integrity and human dignity (2). It encompasses sexual harassment, coercion, assault, exploitation, and emerging forms of Technology-Facilitated Gender-Based Violence (TFGBV). While global and national policies provide a legal foundation for addressing SGBV, their translation into effective institutional practice has remained elusive.

### 1.1 Technology-facilitated gender-based violence (TFGBV)

The digitalization of social and academic life has introduced new dimensions of gender-based violence. Technology-facilitated gender-based violence (TFGBV) refers to harmful behaviors such as cyberstalking, non-consensual sharing of intimate images, cyberbullying, and online grooming. These forms of abuse disproportionately target female students, amplifying their vulnerability in virtual spaces. According to the United Nations Population Fund (3), approximately 85% of women globally have witnessed online violence, and nearly 40% have personally experienced it. In Nigeria, the intensification of such digital abuses contributed to what was described as a “GBV pandemic” in 2020, underscoring the urgent need for interventions addressing both online and offline violence in higher education. As technology becomes increasingly embedded in academic engagement, safeguarding students' digital environments has become integral to ensuring their overall well-being and safety.

### 1.2 Global context

Sexual and gender-based violence (SGBV) is globally recognized as both a public health emergency and a profound violation of human rights. It encompasses acts of physical, sexual, psychological, and economic harm directed at individuals based on gender (4). Globally, an estimated 736 million ever-partnered women aged 15–49 have experienced physical or sexual violence, or both, from an intimate partner at least once in their lifetime (25), while one in three women (30%) have encountered SGBV (3). Sub-Saharan Africa records the highest prevalence rates compared to other regions (5, 26), with evidence showing that SGBV is more widespread in less-developed nations (27).

A meta-analytic review in the Sub-Saharan Africa region identified women's unemployment, religion, low socioeconomic status, and social insecurity as societal factors associated with GBV, while alcohol use, limited education, depression, youthfulness, and a history of abuse were key individual risk factors (6). Similarly, a cross-national study across 27 Sub-Saharan African countries reported that the prevalence of sexual intimate partner violence among adolescent girls and young women aged 15–24 ranged from 6.5% in Comoros to 43.3% in Gabon, with a median of 25.2% (7). Comparable findings indicate a 29% prevalence of GBV among women in East and Southern Africa and a 45% prevalence in Ethiopia (8), underscoring the severity and persistence of the problem in the region. SGBV has far-reaching health and social consequences, including physical injuries, mental health challenges, and long-term social exclusion. Although both men and women may experience SGBV, women and girls bear a disproportionate burden. Adolescent girls and young women are particularly vulnerable to sexual harassment, assault, and exploitation, often within institutions meant to provide protection and empowerment (9). The global persistence of SGBV reflects enduring gender inequalities, entrenched power imbalances, and social norms that perpetuate violence and impunity.

### 1.3 Nigerian tertiary institutions context

In Nigeria, there has been a steady increase in reported sexual violence cases in the country over the years, making Nigerian tertiary institution campuses an unsafe and unhealthy learning environment for students, especially females. In previous empirical studies. Margaret-Mary et al. (28), reported that as high as 46.7% had suffered from one form of sexual violence or the other among female students in respective higher institutions across the South-South geopolitical zone in Nigeria. Although SGBV, particularly sex-for-mark, is a trendy issue globally, its growing prevalence in Nigerian universities and other tertiary institutions is gradually increasing and constituting a source of worry to relevant education stakeholders and human rights advocates (10). It was reported in a recent study that the prevalence of sexual abuse against young women across tertiary institutions within the Federal Capital Territory, Abuja, was 25.3% (11). Apart from this, in 2021, three lecturers of the Obafemi Awolowo University were dismissed over alleged sexual harassment of students. Relatedly, the University of Lagos, the University of Port-Harcourt and Ignatius Ajuru University of Education have equally sacked at least one lecturer based on similar cases (12). The recent public outcry by female law students of the University of Calabar against the Dean of their Faculty, who was later remanded by a Federal High Court over allegations of sexual harassment corroborated the fact that the prevalence of gender-based violence, particularly on Nigerian young women, undoubtedly persists and urgently needs to be addressed (13, 14).

### 1.4 Drivers of SGBV in Nigerian higher education

The persistence of SGBV within Nigerian higher education is deeply intertwined with structural and cultural factors that normalize gender inequality and limit accountability. Patriarchal norms embedded in Nigerian society often position men as authority figures and women as subordinates, reinforcing unequal power relations that are reproduced within academic settings (15, 16). In many universities, lecturer–student hierarchies create environments where male lecturers wield significant control over grades, supervision, and academic progression conditions that can be exploited to solicit sexual favors from female students. This power asymmetry, coupled with the perception of lecturers as “gatekeepers” to success, discourages victims from resisting or reporting abuse (24). Moreover, institutional cultures of silence and stigma further entrench the problem. Victims who report harassment are often disbelieved, blamed, or ostracized by peers and administrators, creating a hostile reporting environment (17, 18). Cultural expectations that demand women's modesty and endurance also shape how SGBV is interpreted and addressed. Within some Nigerian communities, discussions around sex and sexual violence remain taboo, which further discourages open dialogue and advocacy (16). These factors: patriarchal values, entrenched power hierarchies, weak reporting systems, and socio-cultural stigma collectively sustain a cycle of impunity that enables SGBV to thrive within tertiary institutions.

Some efforts have been made to combat SGBV in tertiary institutions through legislative measures such as the Violence against Persons (Prohibition) Act (VAPP) and institutional policies on SGBV. However, enforcement may remain a challenge due to resistance from entrenched institutional cultures that normalize SGBV (24). Also, the role of stakeholders such as Non-Governmental Organizations (NGOs), student advocacy groups has proven vital in bringing gaps in policy implementation and support for victims. Existing studies focus primarily on prevalence rates and legal frameworks but fail to detail the roles played by stakeholders who are involved in the issues of SGBV, their roles, and best practices. Thus, this study aims to identify stakeholders who are involved in the issue of SGBV in Nigerian Tertiary institutions and explore their various roles.

## 2 Theoretical framework

This study was anchored on two interrelated theoretical frameworks: the Socio-Ecological Model (SEM) of violence and the Theory of Change (ToC). Together, these frameworks provide a comprehensive lens for analyzing the multilevel dynamics of Sexual and Gender-Based Violence (SGBV) within Nigerian tertiary institutions and for linking diagnostic insights to actionable interventions.

The Socio-Ecological Model, originally advanced by Bronfenbrenner (19) and later adapted for violence prevention (20), posits that human behavior is shaped by multiple, interacting layers of individual, relational, community, institutional, and societal influences. Applied to SGBV, the model situates incidents of abuse within the broader context of interpersonal relationships, organizational culture, and sociocultural norms. It underscores that SGBV is not merely a product of individual deviance but arises from complex interactions between personal attitudes, institutional practices, and structural inequalities (21).

Within Nigerian tertiary institutions, these dynamics are situated within entrenched patriarchal structures and power hierarchies that normalize gender inequality and silence victims. The SEM helps explain how certain environments—such as campus residences, lecturer–student supervisory relationships, or social gatherings—can become sites where gendered power is exercised and tolerated. This multi-level approach highlights that effective prevention must target not only individual behavior change but also institutional reforms, social norms transformation, and legal accountability mechanisms (22).

While the socio-ecological model diagnoses the layers of risk and influence, the Theory of Change provides a structured framework for designing and sequencing interventions to achieve measurable impact. The ToC translates the problem analysis into a causal pathway linking inputs, activities, and outcomes at different stages of change (23). At the juncture where the SEM identifies leverage points—such as institutional culture, reporting structures, and interpersonal dynamics—the ToC specifies corresponding actions and outcomes. Short-term interventions, such as enhancing reporting channels, training gatekeepers, and providing survivor-centered support services, are expected to yield intermediate outcomes like increased reporting, trust in institutional mechanisms, and improved responsiveness to cases. These cumulative effects are envisioned to culminate in the long-term goal of creating safe and gender-equitable campuses devoid of SGBV.

Integrating the SEM and ToC thus provides both an analytical and action-oriented foundation for this study. Their integration allows the study to move beyond descriptive analyses of SGBV to an explanatory and intervention-oriented framework that links diagnostic insights to actionable pathways for institutional and societal transformation. The socio-ecological model elucidates the nested influences sustaining SGBV in tertiary institutions, while the theory of change offers a logical pathway for transforming these insights into progressive, measurable interventions and policy outcomes. Together, they emphasize that sustainable change requires coordinated efforts across individual, institutional, and structural levels to shift power relations and promote a culture of respect and accountability within higher education spaces. The SEM outlines interventions targeting environmental factors that sustain SGBV, such as the culture of silence, by implementing programs that allow survivors to report abuse without fear of molestation or regret. The ToC will clarify the factors that promote collaborative efforts among stakeholders to develop a safer system.

### 2.1 Gap and study justification

Efforts have been made to address Sexual and Gender-Based Violence (SGBV) in Nigerian tertiary institutions through legislative frameworks such as the Violence Against Persons (Prohibition) Act (VAPP) and various institutional SGBV policies. However, effective enforcement remains a significant challenge, often hindered by deeply entrenched institutional cultures that perpetuate or normalize such violence (24). Tackling this issue requires a coordinated, multi-stakeholder strategy involving government agencies, educational authorities, non-governmental organizations (NGOs), student unions, and advocacy networks. Each stakeholder plays a vital role in prevention, awareness creation, policy implementation, and survivor support. Despite these efforts, existing research in Nigeria has predominantly focused on documenting the prevalence, typologies, and legal frameworks of SGBV, with limited exploration of the specific roles, interactions, and best practices among stakeholders engaged in combating the problem.

Therefore, this study broadens the analytical scope to encompass a cross-institutional perspective that includes federal and state universities, polytechnics, and colleges of education across four Nigerian states. Specifically, it aims to:

1. Identify key stakeholders involved in addressing SGBV within tertiary institutions;
2. Examine their respective roles, patterns of collaboration, and coordination mechanisms; and
3. Highlight effective, contextually relevant practices that have contributed to mitigating SGBV risks within campus environments.

Findings from this study are expected to provide an evidence base for the design of more integrated, sustainable, and institutionally sensitive strategies that promote safer, more equitable, and gender-responsive learning spaces across Nigeria's tertiary education system. Although Technology-Facilitated Gender-Based Violence (TFGBV) is acknowledged as an emerging concern within Nigerian tertiary institutions, it was not a primary focus of the present study and was therefore not explored as a standalone analytical category in the findings.

## 3 Methods and materials

### 3.1 Study design

This study employed a cross-sectional sequential mixed-methods design integrating both quantitative and qualitative approaches to explore Sexual and Gender-Based Violence (SGBV) prevention and response mechanisms in Nigerian tertiary institutions. The quantitative phase generated baseline data on prevalence, awareness, and institutional responses, while the qualitative phase deepened understanding of stakeholder roles, contextual influences, and best practices.

### 3.2 Study locations and justification

The research was conducted across four Nigerian states: Kaduna, Enugu, Osun, and the Federal Capital Territory (FCT). These locations were purposively selected to ensure geographical and institutional diversity, reflecting Nigeria's major geopolitical zones and capturing variations in educational systems and socio-cultural contexts. Kaduna represented the North-West region, Enugu the South-East, Osun the South-West, while the FCT represented the central administrative zone.

### 3.3 Sampling technique

The target population for this study was female and male students who had spent not less than a semester in their respective tertiary institutions. For the quantitative phase, a total of 4,142 students were selected from public and private universities, polytechnics, and colleges of education using a multistage stratified random sampling technique. Among the students in each university (federal and state) (100–500 level), twenty-three (23) female participants and eight (8) male participants were randomly selected in the faculties of arts and sciences, respectively. At the polytechnic, twenty-nine (29) female participants were selected in each of Ordinary National Diploma I and II (OND I and OND II) as well as Higher National Diploma I and II (HND I, and HND II). Lastly, at the colleges of education, 40 female participants and 9 male participants were randomly selected across 100–300 levels.

Within each institution, students were grouped by gender and level of study to ensure broad representation. Female students (*n* = 3,161) were oversampled, given the heightened vulnerability of women to SGBV and the study's focus on gendered experiences.

For the qualitative component, participants were purposively selected based on their institutional roles and relevance to SGBV policy and practice. Key informants included representatives from Federal and State Ministries of Education, National Universities Commission (NUC), National Board for Technical Education (NBTE), law enforcement agencies, Ministry of Justice, Heads of Gender Units, Deans of Student Affairs, Directors of Health Services, Heads of Security Units, student leaders (male and female), members of anti-sexual harassment committees, and Gender Desk Officers of the Nigerian Police serving the participating institutions.

In total, seven Focus Group Discussions (FGDs), twenty-two Key Informant Interviews (KIIs), and twelve In-Depth Interviews (IDIs) were conducted across the study locations. To ensure gender representation, homogeneous FGDs were organized within each institution (one for male and one for female participants) to capture diverse perspectives on institutional culture and response mechanisms to SGBV.

### 3.4 Materials and instruments

Quantitative data were collected using structured, gender-sensitive questionnaires developed from validated SGBV survey tools and adapted to the Nigerian tertiary education context. Separate versions were administered to male and female respondents. The instruments comprised sections on:

1. Socio-demographic characteristics;
2. Institutional layout and welfare systems;
3. Gender norms, roles, and practices;
4. Substance use and risky behaviors;
5. Intimate partner violence and sexual harassment experiences;
6. SGBV awareness, reporting, and safety mechanisms;
7. Institutional capacities and preventive programs.

Each questionnaire was pilot-tested in a tertiary institution outside the study sample to assess clarity, cultural appropriateness, and reliability. Modifications were made based on pilot feedback to improve internal consistency and comprehension.

Qualitative data collection instruments (FGD, KII, and IDI guides) were designed to elicit narratives on institutional culture, campus safety, SGBV experiences, and existing legal and institutional frameworks for prevention and redress. All discussions and interviews were audio-recorded with participants' consent and complemented by detailed field notes.

### 3.5 Data collection and management

Quantitative data were collected using Open Data Kit (ODK) on Android devices, ensuring real-time capture, skip logic enforcement, and minimal entry errors. Data were uploaded daily to a secure, password-protected server and analyzed using Stata statistical software.

Qualitative data (audio files and transcripts) were uploaded daily to encrypted drives, verified for accuracy, and anonymized using pseudonyms to ensure confidentiality. Only authorized research team members had access to the data. All procedures adhered to international data protection and confidentiality standards for sensitive research involving human participants.

### 3.6 Ethical considerations

Ethical clearance for this study was obtained from the Obafemi Awolowo University Institutional Research Board (IRB). Further approvals were secured from participating institutions and relevant government authorities. All participants provided written informed consent after being briefed on the study's purpose, procedures, risks, and benefits. Participation was voluntary, and respondents were informed of their right to withdraw at any point without consequences.

Given the sensitivity of the topic, referral pathways were established with campus counseling units, gender desks, and local non-governmental organizations to support participants who disclosed distress or trauma during data collection.

### 3.7 Data analysis

Descriptive analysis (frequency and percentage) using SPSS version 25.0 was computed on the qualitative data to understand the situation analysis of SGBV in the Nigerian Higher Education Institutions. The analysis of qualitative data followed six steps of thematic analysis, viz: familiarization with the data; generating initial codes; searching for codes in the data; reviewing the themes; defining and naming the themes; and producing the report (Miles and Huberman, 1994; Roberts et al., 2019). To ensure inter-coder reliability, the development of the codebook was done via collaborations, and all the analyses was done with the use of a codebook and analysis matrix which applied to all the transcripts (Roberts et al., 2019). The team ensured quality control during data collection and transcription through various means such as supervisor and qualitative consultant review of interviews and providing real-time feedback to data collectors for improvement. The audio recordings were transcribed verbatim and the resulting transcripts were thoroughly reviewed. Interviews or discussions conducted in indigenous language were translated into English language by language experts. The data was analyzed thematically using ATLAS Version 25, a qualitative analysis software that is specifically designed for the organization, grouping, retrieval and management of qualitative data. The data, which had been transcribed from audio to text documents, was analyzed using both deductive and inductive methods. The analysts first familiarized themselves with the research objectives, research instruments, summary notes, and a few transcripts. They brainstormed and developed deductive codes aligned with the research objectives. The coders shared the codebook and transcripts to perform both deductive and inductive coding. They imported the data into the software and attached the deductively generated codes to the relevant segments of the data. New codes were generated as new issues emerged from the data. The coders discussed these issues periodically to create unified codes. After completing the coding process, the data bundles were combined into a single dataset. The codes were then organized into themes and sub-themes to provide a comprehensive understanding of the phenomena being studied. Finally, the findings were analyzed and discussed thematically based on the classification of codes. The statements made by the informants and interviewees were accurately recorded and grouped into relevant themes and sub-themes to represent their thoughts, emotions, and understanding of the topic being studied. Networks and diagrams were also utilized, where necessary, to visually present the connections and demonstrate the discoveries.

## 4 Results

### 4.1 Respondents' background characteristics

The results in Table 1 present data on respondents' background characteristics based on the type of institution they attend across four different locations: Osun, Enugu, Kaduna, and Abuja. The information is displayed in terms of frequency and percentage for each type of institution.

**Table 1:** Respondents’ background characteristics.

Age	Osun		Enugu		Kaduna		Abuja	
	No.	%	No.	%	No.	%	No.	%
15–19	547	38.7	263	19.4	121	10.2	45	23.7
20–24	719	51.0	942	69.5	740	62.4	115	60.5
25–29	121	8.6	118	44.9	240	20.2	30	15.8
30+	24	1.7	33	3.5	84	7.1	0	0
Total	1,411	100	1,356	100.0	1,185	100.0	190	100
Mean	*21.8*	*20.4*	*22.7*	*21.4*	*24*	*23.0*	*22.8*	*23.0*
Religion								
Christianity	1,067	75.6	1,283	94.6	670	56.5	165	86.8
Islam	341	24.2	35	2.6	503	42.4	25	13.2
Traditional	1	0.7	21	1.5	7	0.6	0	0
Others	2	0.1	17	1.3	5	0.4	0	0
Total	1,411	100.0	1,356	100.0	1,185	100.0	190	100.0
Relationship status								
Currently Married	28	2.0	131	9.0	138	11.6	0	0
Co-Habiting	8	0.6	32	2.2	27	2.3	16	10.1
Girl, not living together	506	35.9	384	26.4	370	31.2	71	44.9
Divorced/separated	0	0	45	3.1	31	2.6	0	0
Widowed	0	0	14	1.0	14	1.2	0	0
No relationship	869	61.6	851	58.4	605	51.1	71	44.9
Total	1,411	100.0	1,356	100.0	1,185	100.0	190	100.0
Type of Residence								
On-Campus	575	40.8	732	53.1	638	53.9	71	37.4
Off-Campus	827	58.6	602	44.4	470	39.7	119	62.6
Both	9	0.6	22	1.6	77	6.5	0	0
Total	1,411	100	1,356	100.0	1,185	100.00	190	100.0
Level in school								
100	338	24.0	200	14.8	103	8.7	48	25.3
200	416	29.5	435	32.1	447	37.7	56	29.5
300	311	22.0	438	32.3	329	27.7	45	23.7
400	284	20.1	190	14.0	262	22.1	41	21.6
500	34	2.4	69	5.1	23	1.9	0	0
600	0	0	0	0	6	0.5	0	0
Postgraduate	28	2.0	23	1.7	16	1.3	0	0
Total	1,411	100.0	1,356	100.0	1,185	100.0	190	100.0
Ethnicity								
Igbo	91	6.4	1,150	84.8	111	9.4	31	16.3
Yoruba	1,269	89.9	104	7.7	239	20.2	37	19.5
Hausa	8	0.6	10	0.7	394	33.2	85	44.7
other	43	3.0	92	6.8	441	37.2	37	19.5
Total	1,411	100	1,356	100	1,185	100.0	190	100.0
Type of Institution								
University	809	57.3	997	73.5	640	54.0	31	16.3
Polytechnic	303	21.5	100	7.4	390	32.9	34	17.9
College of Education	299	21.2	258	19.0	155	13.1	125	65.8
Monotechnic	0	0	**1**	0.1	0	0	0	0
Total	1,411	100.0	1,356	100.0	1,185	100.0	190	100.0

In Osun, 809 respondents (57.3%) are affiliated with a university, indicating a significant presence of university students in the surveyed population. Enugu has the highest percentage of university-affiliated respondents, with 73.5% (997 individuals). This suggests a strong prevalence of university education in Enugu. In Kaduna, 54.0% (640 respondents) are from a university, emphasizing a considerable representation in this category. Abuja, on the other hand, has the lowest percentage of university-affiliated respondents at 16.3% (31 individuals), indicating a smaller presence of university students in the capital city.

Similarly, Osun has 303 respondents (21.5%) attending polytechnics, reflecting a significant proportion of students pursuing polytechnic education. In Enugu, only 7.4% (100 individuals) are from polytechnics, indicating a smaller representation compared to other locations. Kaduna has 32.9% (390 respondents) attending polytechnics, showcasing a substantial presence in this category. Abuja has 17.9% (34 individuals) attending polytechnics, suggesting a moderate representation.

For College of Education, Osun, has 299 respondents (21.2%) enrolled in colleges of education, indicating a notable presence of individuals pursuing education-focused institutions. Enugu has 258 respondents (19.0%) attending colleges of education, representing a significant portion of the surveyed population. Kaduna has 13.1% (155 respondents) attending colleges of education, showing a relatively lower representation compared to other locations. Abuja stands out with 65.8% (125 individuals) attending colleges of education, indicating a predominant focus on education-related institutions. Conversely, at the time of data collection, monotechnic institutions in Osun State were not in session, which limited access to students and accounts for the absence of respondents from monotechnics in that state, In Enugu, only 0.1% (1 individual) attends a mono-technic, while there are no respondents from Kaduna or Abuja attending mono-technics. Universities dominate the educational scene in Enugu, Osun, and Kaduna, with substantial percentages of respondents affiliated with these institutions. However, Abuja deviates from this trend, with a significantly lower percentage attending university. Polytechnics have a considerable presence in Osun and Kaduna, while colleges of education are notably represented in Osun, Enugu, and Abuja. This diversity suggests varying educational priorities and preferences among respondents in different locations. Abuja stands out as having a unique educational landscape, with a relatively lower percentage of university attendees but a predominant focus on colleges of education. This could be influenced by the city's role as the capital and its emphasis on education-related institutions. Monotechnics have minimal representation across all locations, indicating that respondents in this survey are primarily enrolled in universities, polytechnics, or colleges of education.

#### 4.1.1 Key stakeholders involved in addressing SGBV in Nigerian tertiary institutions

**Figure 1:**
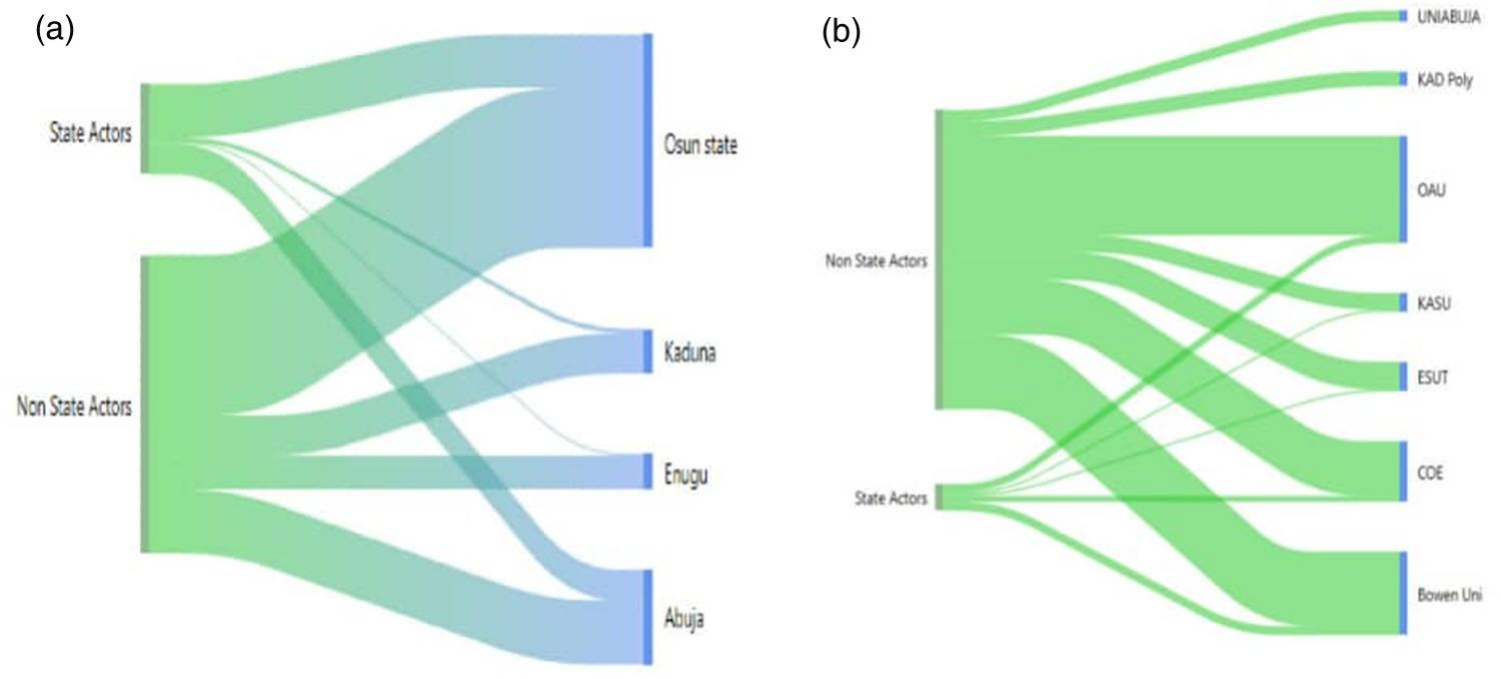
**(a)** Sankey diagram showing the density of state and non-state actors by state. **(b)** Sankey diagram showing the density of state and non-state actors by institution.

The study identified a diverse range of stakeholders involved in addressing Sexual and Gender-Based Violence (SGBV) across the surveyed tertiary institutions. These stakeholders were classified into two broad categories to include state actors and non-state actors. The frequency distribution of participants' responses across states and institutions are presented in Table 2 and illustrated in Figures 1a,b.

**Table 2:** Distribution of participants’ responses on identified SGBV stakeholders by state and institution.

Stakeholder Category	Osun	Enugu	Kaduna	FCT (Abuja)	Total Responses (n)	Percentage (%)	Most Cited Institutions
Non-State Actors							
Academic Staff	280	40	40	120	480	11.6	OAU, Bowen Univ., UNIABUJA
Parents	240	80	80	120	520	12.6	OAU, Bowen Univ., COED Abuja
Religious Bodies	160	40	80	80	360	8.7	OAU, ESUT
Community Leaders	120	40	80	40	280	6.8	KASU, COED Abuja
Student Associations/Unions	320	120	120	160	720	17.4	OAU, UNIABUJA, KASU
Non-Governmental Organizations (NGOs)	240	80	120	120	560	13.5	OAU, KASU, ESUT
Non-Academic Staff	200	80	80	120	480	11.6	OAU, Kaduna Poly
Subtotal Non-State Actors	1,560 (37.7%)	480 (11.6%)	600 (14.5%)	760 (18.3%)	3,400	82.1	–
State Actors							
Ministry of Education	160	0	80	120	360	8.7	KASU, COED Abuja, OAU
Law Enforcement Agencies	120	40	80	80	320	7.7	UNIABUJA, KASU
Judiciary/Ministry of Justice	80	0	40	80	200	4.8	KASU, COED Abuja
School Management	120	40	80	80	320	7.7	OAU, UNIABUJA, Kaduna Poly
Subtotal State Actors	480 (11.6%)	80 (1.9%)	280 (6.8%)	360 (8.7%)	1,220	28.9	–
Total (All Stakeholders)	2,040 (49.3%)	560 (13.5%)	880 (21.2%)	1,120 (27.0%)	4,142 (100%)	100	–

Most cited institutions: OAU, Obafemi Awolowo University; UNIABUJA, University of Abuja; KASU, Kaduna State University; COED Abuja, College of Education, Abuja; ESUT, Enugu State University of Science and Technology; Bowen University (private).

As shown in Table 2, non-state actors constituted the majority of stakeholders mentioned (≈82%), underscoring their centrality in campus-based SGBV prevention and response. Key non-state contributors included student associations (17.4%), parents (12.6%), academic staff (11.6%) and NGOs (13.5%). These groups were described as the most visible and proactive across all study sites, particularly in Osun State (37.7%) and the Federal Capital Territory (FCT) (18.3%). In contrast, state actors, including the Ministry of Education (8.7%), law enforcement agencies (7.7%), the judiciary (4.8%), and school management (7.7%), were primarily recognized for policy formulation, enforcement, and institutional oversight roles. Collectively, state actors accounted for 28.9% of total mentions, reflecting their formal authority but limited operational visibility at the institutional level.

Qualitative data reinforced these patterns, with participants consistently highlighting the dominance of non-state actors, particularly NGOs and student-led organizations**,** in driving SGBV awareness, advocacy, and survivor support initiatives on campuses.

Most of the awareness and support programs we see on campus come from NGOs and student groups, not the government. They are the ones organizing campaigns and giving students a voice. (FGD, Female Student, OAU)

This sentiment was echoed across study sites, where respondents described NGOs and student unions as the most visible drivers of preventive interventions, often filling gaps left by institutional or governmental structures.

Whenever there is a gender-related campaign, it usually comes from outside organizations or our student union. Government agencies rarely visit or follow up. (FGD, Male Student, Kaduna State University)

NGOs have done more to sensitize students about sexual harassment than any government body. They talk to us in a way we can understand. (FGD, Female Student, Enugu State University of Science and Technology)

Participants further emphasized the peer-led and culturally resonant nature of student association campaigns, which often used debates, theatrical performances, posters, and social media platforms to raise awareness and normalize conversations about gender-based violence.

When we hear fellow students speak about these issues in drama or during campaigns, it feels real and makes more people come forward. (IDI, Female Student Leader, University of Abuja)

Across all institutions, non-state actors were consistently perceived as more accessible, responsive, and community-oriented, while formal state mechanisms were described as “distant,” “bureaucratic,” or “too official” to connect with students' lived realities.

Government officers only come during official visits; they don't understand what really happens on campus. But NGOs stay in touch with students and help even after cases occur. (KII, Student Affairs Officer, Osun State)

Several institutional representatives acknowledged that NGOs often serve as a bridge between students and authorities, offering immediate support in cases where school or government response systems were slow or uncoordinated.

When cases are reported, NGOs are the first to respond or refer victims for help. They are faster and more empathetic than school or government channels. (KII, Gender Desk Officer, FCT)

Quantitative mapping further corroborated these perceptions, revealing that NGOs, student bodies, and parents recorded the highest density of involvement across all institutions, while the school management remained the most frequently cited state stakeholder based on their direct engagement in day-to-day SGBV prevention and response.

### 4.2 Roles, collaboration patterns, and coordination mechanisms among stakeholders

Findings from both the quantitative and qualitative strands revealed that stakeholders in tertiary institutions perform distinct yet interrelated roles in preventing and responding to SGBV. These roles are shaped by their institutional mandates, resources, and levels of engagement with students and staff. Tables 3a,b present the distribution of respondents' perceptions of stakeholder roles across the four study states based on stakeholders' categorie.

**Table 3a:** Frequency distribution of non-state actors’ roles in addressing SGBV across tertiary institutions by state (*N* = 4,142).

Stakeholder Category	Key Roles and Responsibilities	Osun	Enugu	Kaduna	FCT (Abuja)	Total Responses (n)	Percentage (%)
Non-State Actors							
Non-Governmental Organizations (NGOs)	Awareness creation, survivor counseling, advocacy, and legal aid	270	80	100	130	580	14.0
Student Associations/Unions	Peer education, reporting, mobilization, advocacy, and liaison with management	310	100	120	160	690	16.6
Academic Staff	Mentorship, ethics enforcement, policy advocacy, and protection of students	250	80	120	100	550	13.3
Non-Academic Staff	Security oversight, early reporting, and welfare monitoring	210	80	90	100	480	11.6
Parents	Moral upbringing, counseling, and family advocacy for victims	230	70	80	100	480	11.6
Religious & Community Leaders	Value re-orientation, mediation, and cultural advocacy	180	60	70	80	390	9.4
Subtotal Non-State Actors		1,450 (35.0%)	470 (11.4%)	580 (14.0%)	670 (16.2%)	3,170	≈76.6

#### 4.2.1 Roles of non-state actors

Non-state stakeholders were consistently portrayed as the most visible, responsive, and trusted agents in SGBV prevention and response, as depicted in Table 3a.

**Figure 2:**
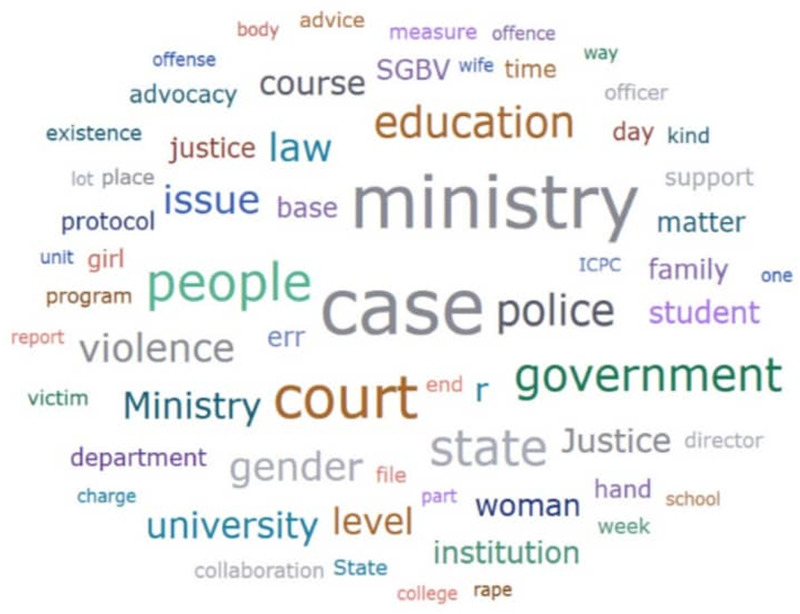
The role of non-governmental organisations (NGOs).

NGOs were consistently identified as the most visible non-state players, providing services that include peer education, psychosocial counseling, legal aid, and community sensitization (Figure 2). Their programs often filled the gaps left by weak institutional structures. One NGO representative described this role succinctly:

We organize sensitization sessions and train student leaders on how to identify and support survivors. In most cases, we also provide counseling and connect victims with legal help. (KII, NGO Representative, Abuja)

Student associations and unions were recognized as strong peer advocates, acting as the link between management and students. They organize campaigns, debates, and workshops aimed at reshaping gender norms and encouraging bystander interventions.

We hold our own awareness weeks and use social media to educate others. Students trust their peers more than formal channels when it comes to reporting cases. (FGD, Male Student Leader, Kaduna)

**Figure 3:**
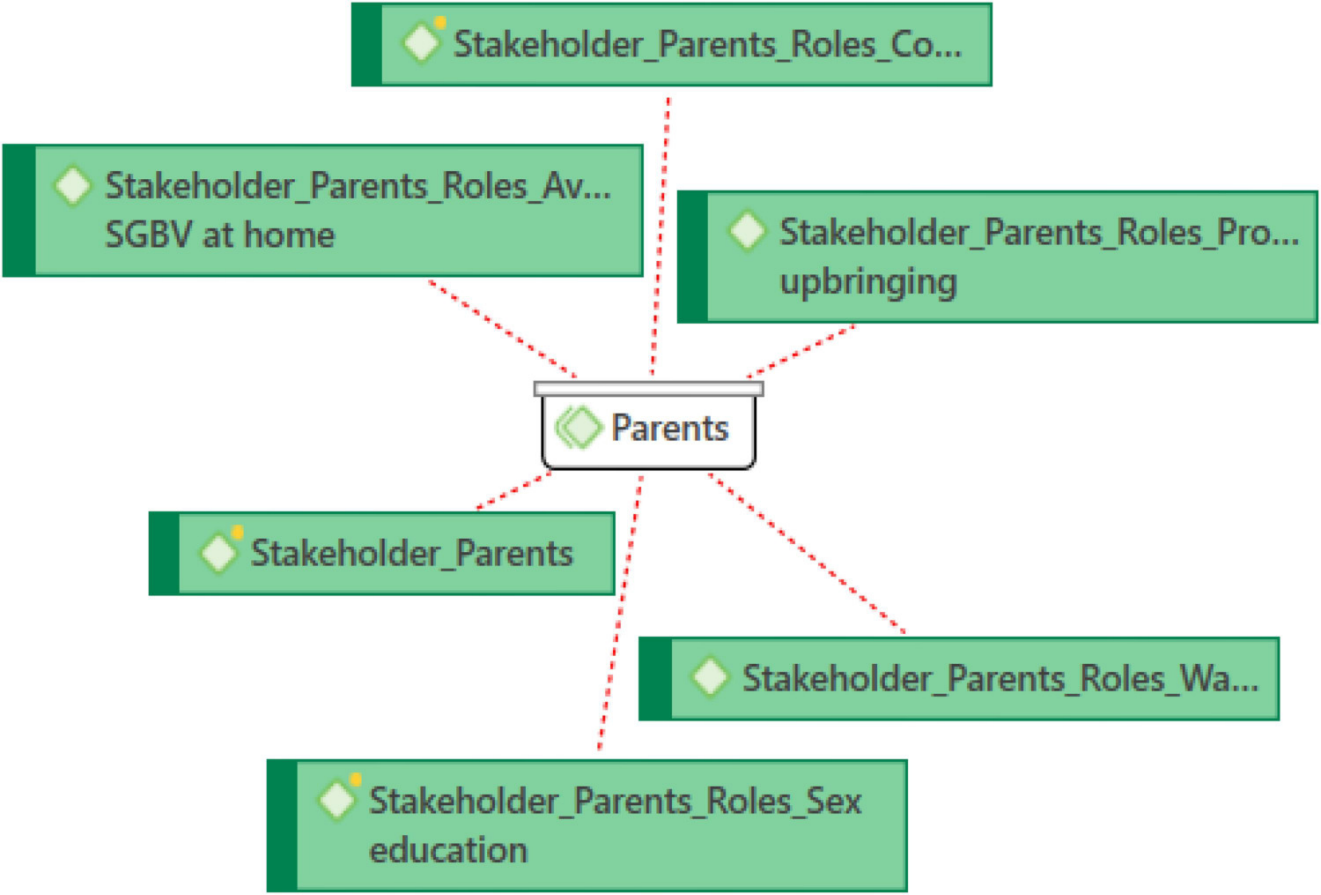
The Roles of Parents as Stakeholder.

Parents and religious leaders were seen as important influencers of moral behavior and social values, while academic and non-academic staff were described as “custodians of ethics” who are expected to protect students and model professional conduct (Figure 3). However, some participants noted that inconsistent staff behavior sometimes undermines these expectations.

#### 4.2.2 Roles of state actors

The result in Table 3b shows the level of state actors' involvement in the issue of SGBV in tertiary institutions

**Table 3b:** Frequency distribution of state actors’ roles in addressing SGBV across tertiary institutions by state (*N* = 4,142).

Stakeholder Category	Key Roles and Responsibilities	Osun	Enugu	Kaduna	FCT (Abuja)	Total Responses (*n*)	Percentage (%)
Ministry of Education	Policy formulation, supervision, and awareness collaboration	160	0	80	100	340	8.2
Law Enforcement Agencies	Investigation, enforcement, referral, and protection	130	40	80	90	340	8.2
Judiciary/Ministry of Justice	Legal adjudication, prosecution, and sanctioning of offenders	70	0	40	70	180	4.3
School Management	Policy enforcement, complaint handling, disciplinary oversight, and institutional coordination	150	50	70	80	350	8.4
Subtotal State Actors		510 (12.3%)	90 (2.2%)	270 (6.5%)	340 (8.2%)	1,210	≈29.2
Total (All Stakeholders)		1,960 (47.3%)	560 (13.5%)	850 (20.5%)	1,010 (24.4%)	4,142 (100%)	100.0

State actors, including the Ministry of Education, law enforcement agencies, the judiciary, and the school management, were found to be mainly responsible for policy formulation, regulatory oversight, and law enforcement. Participants highlighted that although relevant policies exist, implementation is inconsistent across institutions. Illustratively:

The policies are there on paper, but there is little enforcement. Universities need to integrate these laws into their own disciplinary systems. (KII, Ministry of Education Official, Kaduna)

Law enforcement agencies, especially the Nigeria Police Force Gender Desk, were acknowledged for responding to severe cases but were criticized for slow investigation processes and inadequate sensitivity in handling survivors' experiences. Some respondents emphasized the need for capacity building among security personnel and for stronger linkages between police and institutional gender desks.

#### 4.2.3 Stakeholders' collaboration and coordination mechanisms

Qualitative findings revealed that collaboration among stakeholders in addressing Sexual and Gender-Based Violence (SGBV) within tertiary institutions was limited, largely informal, and often event-driven rather than sustained. While most institutions acknowledged the presence of multiple actors working to prevent or respond to SGBV, structured coordination frameworks were either weak or absent. Partnerships were more visible between Non-Governmental Organizations (NGOs) and student bodies, and between NGOs and gender units, than between tertiary institutions and government agencies such as the Ministry of Education, law enforcement, or the judiciary. Illustratively.

We collaborate with NGOs whenever they come to train our students or staff, but there is no standing committee that brings everyone together regularly. (IDI, Gender Desk Officer, Osun)

NGOs and student unions work closely because they understand student issues better. When government agencies come, it's usually for one-time campaigns. (FGD, Male Student Leader, Kaduna State University)*

There is little follow-up after these awareness programs. Once the project ends, everything stops until another organization comes. (KII, Administrative Officer, Enugu State University of Science and Technology)

Across most study locations, cross-sectoral collaboration tended to occur only during donor-funded projects or advocacy campaigns, rather than through a sustained institutionalized structure. Participants consistently highlighted that while short-term partnerships generated visibility and awareness, their episodic nature limited continuity and impact.

We see a lot of activities when NGOs get funding, but when the project finishes, the connection between them and the school disappears. (FGD, Female Student, University of Abuja)*

Government agencies don't have regular contact with us. It's the NGOs that follow up or call meetings when something serious happens. (KII, Gender Desk Officer, Kaduna Polytechnic)

The absence of a formal coordination mechanism was identified as a major barrier to effective SGBV response. Respondents reported duplication of efforts, an inconsistent referral system, and fragmented institutional responses, especially in cases requiring legal follow-up or psychosocial support.

Sometimes, two different NGOs handle the same case without knowing. There's no platform where everyone updates each other on what they are doing. (FGD, Female Student, Osun State)

Victims are referred from one office to another without coordination. Some lose interest and withdraw their complaints because the process is confusing. (IDI, Female Counselor, Enugu)*

Despite these challenges, positive examples of functional collaboration were observed, particularly in Osun and the Federal Capital Territory (FCT). In these locations, more structured partnerships between gender units, student associations, and NGOs contributed to improved awareness, smoother referral systems, and stronger survivor support.

We have a partnership with an NGO that trains our peer educators and connects us with legal aid when needed. It has made reporting easier. (KII, Student Affairs Officer, University of Abuja)

In Osun, the gender desk works with a local NGO to manage cases. *They share information and organize joint awareness programs every semester. (FGD, Female Student, OAU)*

Having different groups work together has made a big difference here. Students now know where to report, and cases are handled faster. (IDI, NGO Representative, FCT)

These collaborative initiatives, though limited in scale, demonstrated that institutionalized, multi-stakeholder coordination can significantly enhance campus safety, improve referral pathways, and ensure timely responses to SGBV incidents. However, such examples remain exceptions rather than the norm, underscoring the need for a structured, nationwide coordination framework that integrates all key actors and aligns their roles for sustainability.

Overall, the findings underscore that while state actors possess the legal and institutional authority to regulate and enforce anti-SGBV measures, their impact is constrained by weak coordination, resource limitations, and institutional inertia. Enhanced inter-agency collaboration, especially between ministries, law enforcement, and tertiary institution management, remains essential to achieving a coherent, survivor-centered response framework.

### 4.3 Institutional policies and coordination gaps

Institutional audits revealed significant variation in the presence and implementation of anti-SGBV policies across the studied institutions. Only a few institutions had explicit policies, such as anti-sexual harassment frameworks or formal Codes of Conduct, while others relied on student handbooks or general disciplinary guidelines. Table 4 summarizes the availability of such documents across the selected institutions.

**Table 4:** SGBV policies available by institutions selected for the qualitative study.

Anti-SGBV policies	Bowen	COED Abuja	ESUT	KAD Poly	KASU	OAU	UNIABUJA	Total
Anti-Sexual harassment Policy	0	0	0	0	0	6	0	**6**
Code of conduct	0	3	0	0	1	5	1	**10**
Handbook	7	3	0	0	0	2	2	**14**
SGBV Acts/Laws	2	2	0	0	0	4	0	**8**
Total	**9**	**8**	**0**	**0**	**1**	**17**	**3**	**38**

Participants across focus groups emphasized that even where policies existed, they were poorly disseminated and rarely implemented:

There is a policy, but many students don't even know it exists. Some staff members, too, are not aware of it. (FGD, Female Student, Enugu)

The issue is not having policies; it's the silence when cases happen. Students fear reporting because nothing happens afterward. (IDI, Student Leader, Kaduna)

This limited awareness and weak enforcement reflect institutional cultures that often normalize abuse or silence victims, consistent with prior findings on higher education in Nigeria.

Interestingly, most of the available policies across the selected institutions did not address Technology-facilitated sexual and gender-based violence (T-FGBV). This might be associated with the fact that T-FGBV is an emerging form of GBV. A major policy gap is therefore the need to update institutional based GBV policies to reflect the emerging trends and develop robust policies, support services, and awareness campaigns to protect students and nip the menace of GBV in the bud.

### 4.4 Effective and contextually relevant practices for mitigating SGBV

Despite these structural limitations, several best practices emerged across states and institutions:

1. Campus–NGO Collaboration: Partnerships between student unions and NGOs, particularly in Osun and Abuja, have resulted in awareness campaigns, gender safety clubs, and reporting hotlines. *Our NGO trains peer counselors and works with student leaders to identify and support victims discreetly. (KII, NGO Representative, Osun)*
2. Students-Led Advocacy: Student associations have increasingly organized peer sensitization drives, debates, and theatre-based education to challenge cultures of silence. *When students educate each other, it breaks the fear barrier. People start to talk about what they've experienced. (FGD, Male Student, FCT)*
3. Institutional Disciplinary Committees: Institutions like Obafemi Awolowo University were recognized for having Sexual Harassment Committees that handle formal complaints and enforce sanctions against perpetrators. *The committee in our school now takes these issues seriously. Some lecturers have even been dismissed. (IDI, Academic Staff, Osun)*
4. Psychosocial Support Systems: Some Student Affairs Divisions have integrated counseling units and anonymous helplines for emotional support and referrals.
5. Media and Technology-Based Advocacy: NGOs and student influencers increasingly utilize social media platforms to raise awareness and counter Technology-Facilitated Gender-Based Violence (TFGBV).

Although these practices have demonstrated promise, participants emphasized that institutional support and government involvement remain essential for sustaining and scaling up these initiatives.

## 5 Discussion

This article examined the functions of stakeholders and pinpointed best practices for tackling SGBV in Nigerian higher education institutions, aiming to inform effective policy and intervention strategies. The research that underpins this article is among the few national studies employing mixed methods (both qualitative and quantitative) to explore the roles of stakeholders in managing SGBV within Nigerian tertiary institutions. Furthermore, the study's population encompassed a range of tertiary institutions, including universities, colleges of education, and polytechnics. The research offers a distinctive contribution to the existing body of literature by specifically addressing significant gaps through an inclusive, cross-institutional approach to analyze SGBV prevention and response mechanisms across various tertiary institutions in Nigeria.

The findings revealed a diverse network of stakeholders engaged in addressing Sexual and Gender-Based Violence (SGBV) within Nigerian tertiary institutions, broadly classified into state and non-state actors. Across all study locations, non-state actors emerged as the most visible and influential in both preventive and response efforts. Among these, student associations, higher institution staff, parents, and non-governmental organizations (NGOs) were most frequently identified as key actors in shaping institutional and community-level responses.

Higher institution staff comprising both academic and non-academic personnel were recognized as pivotal in modeling institutional culture and shaping students' attitudes toward gender relations. Their expected roles included campaigning against all forms of SGBV, providing counseling and moral guidance, supporting survivors, and maintaining open, empathetic relationships with students. These responsibilities align with the socio-ecological model, which underscores the influence of interpersonal and institutional factors in shaping behavioral outcomes. As role models and authority figures, staff members are positioned at the mesosystem level where power dynamics and moral standards directly affect students' experiences. Strengthening these roles is therefore crucial in addressing practices such as “sex-for-grades” and other forms of abuse that remain pervasive in some Nigerian tertiary institutions (10, 28).

Participants also identified parents as essential stakeholders. Parents constitute the foundational socializing agents in a child's life and are instrumental in the early development of values, self-esteem, and gender attitudes. Participants' views highlight that effective parental upbringing can significantly reduce susceptibility to abusive relationships and normalize respectful interpersonal behavior. This finding aligns with UNICEF (9), which emphasizes that early childhood socialization and parental influence play crucial roles in shaping responses to gender norms and violence. Within the logical theory of change, parental engagement represents an upstream intervention point, where foundational value transmission can yield long-term preventive effects across generations.

Furthermore, NGOs were highlighted as key actors in awareness creation, counseling, empowerment, financial support for law enforcement agencies, and advocacy against sexual violence. However, participants observed that NGO activities were unevenly distributed across the study sites—seven identified in Osun, three in Abuja, and only one each in Kaduna and Enugu. This uneven spread suggests a geographic and operational imbalance that may hinder the sustainability of national-level SGBV responses. The finding corroborates earlier reports that NGO interventions are often concentrated in urban or politically active areas, leaving rural or less visible regions underserved (13, 14). Within the socio-ecological framework, this limited reach constrains community-level mobilization, while in the logic model, it reflects a gap between planned intervention inputs and desired behavioral or policy-level outcomes. Addressing this imbalance requires coordinated strategies among NGOs, governmental agencies, and tertiary institutions to ensure equitable distribution of SGBV prevention and response programs nationwide.

The Ministries and school management are state actors that were mentioned by participants. The ministry is a state actor with direct links to the Government. Only participants from three states identified the Ministry as one of the prominent stakeholders in the fight against sexual and gender-based violence. The majority of the participants who identified Government ministries as one of the stakeholders in the fight against SGBV were from Osun state and Abuja. The third state is Kaduna. The participants from Enugu did not identify Government Ministries as stakeholders in the fight against SGBV. At the institutional level, only participants from Kaduna State University and the College of Education, Abuja, identified Government Ministries as stakeholders in relation to SGBV. The presence of many non-state actors may also show that while national efforts in combating SGBV are limited, non-state actors are allowed to operate thus complementing the efforts of state actors. State actors are essential in enforcing legal frameworks and implementing SGBV-related policies; their role was reported to be less visible compared to non-state actors. This aligns with research suggesting that despite the existence of legislative tools such as the Violence Against Persons (Prohibition) Act, enforcement remains weak due to institutional resistance and normalization of abuse within higher institutions (Ibrahim & Abubakr, 2022; 12).

### 5.1 Power and policy context

The findings revealed limited visibility of state actors in the SGBV response landscape within tertiary institutions. Government ministries, law enforcement agencies, and judicial bodies were often mentioned less frequently than NGOs and campus-based organizations. This disparity reflects broader systemic challenges such as weak political commitment, inadequate funding, and bureaucratic inertia that impede the implementation of national legal frameworks like the Violence Against Persons (Prohibition) Act (VAPP, 2015).

Participants described government-led interventions as sporadic and project-based rather than continuous. Ministries were said to appear mainly during official campaigns or policy launches, with little sustained engagement afterward. Law enforcement agencies were acknowledged for their statutory roles in investigation and prosecution but were often criticized for slow responses and inadequate survivor sensitivity. These observations are consistent with earlier findings that the gap between policy formulation and implementation in Nigeria remains wide, particularly in the education sector (Ibrahim & Abubakr, 2022; 12).

Furthermore, gendered hierarchies within academic institutions whereby men dominate leadership and decision-making structures were identified as major barriers to policy enforcement and survivor protection. Such patriarchal arrangements perpetuate silence, minimize accountability, and reinforce cultures of victim-blaming. This underscores that SGBV in tertiary institutions is not only a behavioral issue but also a symptom of entrenched gendered power relations that sustain inequality.

The findings foreground a paradox central to SGBV scholarship in the Global South—the outsourcing of state responsibility to non-state actors. While NGOs, student associations, and parents emerged as the primary agents of prevention and advocacy, their prominence exposes the fragility of state-led institutional accountability. This imbalance mirrors what feminist institutionalists have termed the “delegated governance of gender justice,” where civil society fills policy vacuums left by weak or complicit state structures. In this sense, Nigeria's tertiary education landscape exemplifies a broader African trend in which anti-SGBV responses depend on donor-driven or episodic activism rather than embedded institutional reform.

Moreover, the findings deepens the ecological understanding of SGBV by empirically demonstrating how individual vulnerabilities intersect with institutional inertia and patriarchal power hierarchies. The limited enforcement of the Violence Against Persons (Prohibition) Act and the patchy institutional adoption of anti-harassment policies reveal that legislation without transformation of institutional culture remains ineffectual. This aligns with global feminist critiques that emphasize that gender violence persists not from policy absence but from “cultural legitimization and bureaucratic complicity” (2).

### 5.2 Stakeholder roles and collaboration patterns

Across study locations, non-state actors were found to perform the majority of SGBV prevention and response functions. NGOs and student associations were particularly active in peer education, legal aid linkage, counseling, and advocacy campaigns, while parents and religious leaders contributed to value reorientation and moral guidance. Academic and non-academic staff served as role models and moral anchors within the institutional environment, although participants noted that inconsistent role performance sometimes diminished their credibility.

By contrast, state actors such as ministries and law enforcement agencies were primarily associated with formal oversight, policy development, and disciplinary enforcement. However, their engagement was perceived as inconsistent and often limited to selected institutions or regions. The absence of continuous collaboration among stakeholders was a recurring theme. Partnerships between NGOs, gender desks, and student unions tended to be event-based—often emerging during donor-funded projects or specific awareness campaigns—and rarely institutionalized. In locations such as Osun and the Federal Capital Territory (FCT), where more structured partnerships existed, participants reported greater awareness, better coordination, and faster case resolution. These cases demonstrate the potential of sustained multi-stakeholder collaboration to strengthen institutional responses and create safer academic spaces.

### 5.3 Institutional policy environment

Institutional audits revealed marked variation in the existence and implementation of anti-SGBV frameworks. Only a few institutions had comprehensive policies, including anti-sexual harassment frameworks and gender-specific disciplinary committees. Many relied on general Codes of Conduct or student handbooks, which were either outdated or poorly disseminated. Participants repeatedly mentioned that students and even some staff members were unaware of the existence of SGBV-related policies, while survivors often lacked clarity about reporting channels. The inadequate awareness, coupled with fear of retaliation, may have contributed to widespread underreporting. These patterns reflect findings from earlier studies that documented weak enforcement and cultural normalization of abuse in Nigerian tertiary institutions (3, 28). In addition, most of the institutional policies did not adequately address TFGBV, despite being an emerging form of, with its prevalence (3).

### 5.4 Regional variations

The findings on Regional differences were apparent across the four study locations. States such as Osun and the Federal Capital Territory displayed stronger multi-actor collaboration, with more active NGO engagement and visible institutional policies. In contrast, Kaduna and Enugu exhibited weaker coordination, fewer active stakeholders, and limited policy implementation. These variations likely mirror broader socio-political and cultural contexts where civil society strength, government commitment, and local attitudes toward gender norms differ considerably. Understanding these contextual influences is essential for designing responsive, region-specific strategies to combat SGBV.

By extending the ToC framework to stakeholder collaboration, the study reveals that sustainable change in tertiary institutions requires structured, interdependent mechanisms—bridging government authority, institutional responsibility, and community participation. The regional variations observed between Osun/FCT and Enugu/Kaduna further highlight how socio-political contexts and civil society density mediate the success of anti-SGBV strategies. This comparative insight adds an important dimension to African SGBV discourse, demonstrating that interventions must be culturally and regionally adaptive rather than uniform.

### 5.5 Implications for policy and practice

The findings highlight the urgent need for systemic reforms that move beyond reactive interventions toward sustainable, coordinated approaches. Tertiary institutions should be required to establish and enforce comprehensive anti-SGBV frameworks, supported by regular staff training and continuous student awareness programs. State ministries and government agencies must play stronger oversight roles by ensuring that national laws such as the VAPP Act are operationalized at the institutional level. Collaboration between state and non-state actors should be formalized through standing committees or task forces to enhance coordination and ensure continuity beyond short-term projects. Equally important is the need to address gendered power asymmetries within universities by promoting women's representation in leadership, integrating gender sensitivity into curricula, and cultivating campus cultures that prioritize respect, equity, and accountability. Ultimately, the study challenges the normative assumption that policy presence equates to institutional safety, emphasizing instead that cultural transformation and shared accountability are the foundations of a violence-free academic environment.

### 5.6 Conclusion

The study reveals that while non-state actors have become the driving force behind SGBV prevention and response within Nigerian tertiary institutions, their impact is constrained by fragmented coordination and minimal state engagement. Building a coherent, sustainable response requires stronger political commitment, institutional accountability, and gender-transformative strategies that dismantle entrenched power hierarchies and create safer, more inclusive academic environments.

## Data Availability

The raw data supporting the conclusions of this article will be made available by the authors, without undue reservation.
