# Supplementary material for: Sexual and gender based violence in Nigeria tertiary institutions: exploring the roles of stakeholders and best practices
Source: Front Glob Womens Health. 2026 May 13;7:1700008. doi: 10.3389/fgwh.2026.1700008 (PMC13212308; doi:10.3389/fgwh.2026.1700008)
Supplement: Supplementary file 1 [file Datasheet1.docx]

**Table 1: Roles being played and Expected roles to be played by School Management**

|  | **Bowen** | **COED Abuja** | **ESUT** | **KAD Poly** | **KASU** | **OAU** | **UNIABUJA** | **Total** |
| --- | --- | --- | --- | --- | --- | --- | --- | --- |
| **Roles being played by School Management** | | | | | | | | |
| Punishing the offenders | 2 | 2 | 2 | 1 | 0 | 5 | 1 | **13** |
| Counselling | 2 | 2 | 0 | 2 | 0 | 4 | 2 | **12** |
| Sensitization | 4 | 4 | 1 | 0 | 0 | 7 | 4 | **20** |
| Policy | 3 | 0 | 0 | 0 | 0 | 6 | 0 | **9** |

**Table 2: NGOs Listed by Participants by State**

| **S/N** | **NGOs** | **Osun** | **Kaduna** | **Enugu** | **Abuja** |
| --- | --- | --- | --- | --- | --- |
| 1 | National Democratic Institute (NDI) |  |  |  |  |
| 2 | PAL |  |  |  |  |
| 3 | ASIDO Foundation |  |  |  |  |
| 4 | Campus Help and Rights Initiative |  |  |  |  |
| 5 | ICPC |  |  |  |  |
| 6 | ALAC |  |  |  |  |
| 7 | Medeciens Sans Frontier (MSF) |  |  |  |  |
| 8 | Yan Sakai |  |  |  |  |
| 9 | War Affected Peoples Association (WAPA) |  |  |  |  |
| 10 | Covenant Orphanage Homes |  |  |  |  |
| 11 | Abraham Orphanage Home |  |  |  |  |
| 12 | Women Against Rape, and Sexual Harassment and Sexual Exploitation (WARSHE) |  |  |  |  |
| 13 | Break the Silence, Act Now! |  |  |  |  |
| 14 | United Nations Population Fund (UNFPA) |  |  |  |  |

**Table 3: SGBV Policies Available by Institutions selected for the Qualitative study.**

|  | **Bowen** | **COED Abuja** | **ESUT** | **KAD Poly** | **KASU** | **OAU** | **UNIABUJA** | **Total** |
| --- | --- | --- | --- | --- | --- | --- | --- | --- |
| **Anti-Sexual harassment Policy** | 0 | 0 | 0 | 0 | 0 | 6 | 0 | **6** |
| **Code of conduct** | 0 | 3 | 0 | 0 | 1 | 5 | 1 | **10** |
| **Handbook** | 7 | 3 | 0 | 0 | 0 | 2 | 2 | **14** |
| **SGBV Acts/Laws** | 2 | 2 | 0 | 0 | 0 | 4 | 0 | **8** |
| **Total** | **9** | **8** | **0** | **0** | **1** | **17** | **3** | **38** |
